# Efficient Toluene Decontamination and Resource Utilization through Ni/Al_2_O_3_ Catalytic Cracking

**DOI:** 10.3390/molecules29204868

**Published:** 2024-10-14

**Authors:** Yifei Niu, Xiaolong Ma, Guangyi Lu, Dandan Zhao, Zichuan Ma

**Affiliations:** 1 Hebei Key Laboratory of Inorganic Nano-Materials, College of Chemistry and Material Sciences, Hebei Normal University, Shijiazhuang 050024, China; niuyf@stu.hebtu.edu.cn (Y.N.); luguangyi1838@163.com (G.L.); zhaodd@stu.hebtu.edu.cn (D.Z.); 2 School of Environmental Science and Engineering, Hebei University of Science and Technology, Shijiazhuang 050018, China

**Keywords:** VOCs, Ni/Al_2_O_3_ catalyst, toluene pyrolysis, CO_x_-free hydrogen, carbon nanofibers

## Abstract

Volatile organic compounds (VOCs), particularly aromatic hydrocarbons, pose significant environmental risks due to their toxicity and role in the formation of secondary pollutants. This study explores the potential of catalytic pyrolysis as an innovative strategy for the effective remediation and conversion of aromatic hydrocarbon pollutants. The research investigates the high-efficiency removal and resource recovery of the VOC toluene using a Ni/Al_2_O_3_ catalyst. The Ni/Al_2_O_3_ catalyst was synthesized using the impregnation method and thoroughly characterized. Various analytical techniques, including scanning electron microscopy, X-ray diffraction, and N_2_ adsorption–desorption isotherms, were employed to characterize the Al_2_O_3_ support, NiO/Al_2_O_3_ precursor, Ni/Al_2_O_3_ catalyst, and the resulting solid carbon. Results indicate that Ni predominantly occupies the pores of *γ*-Al_2_O_3_, forming nano/microparticles and creating interstitial pores through aggregation. The catalyst demonstrated high activity in the thermochemical decomposition of toluene into solid carbon materials and CO_x_-Free hydrogen, effectively addressing toluene pollution while recovering valuable resources. Optimal conditions were identified, revealing that a moderate temperature of 700 °C is most favorable for the catalytic process. Under optimized conditions, the Ni/Al_2_O_3_ catalyst removed 1328 mg/g of toluene, generated 915 mg/g of carbon material, and produced 1234 mL/g of hydrogen. The prepared carbon material, characterized by its mesoporous structure and high specific surface area graphite nanofibers, holds potential application value in adsorption, catalysis, and energy storage. This study offers a promising approach for the purification and resource recovery of aromatic volatile organic compounds, contributing to the goals of a circular economy and green chemistry.

## 1. Introduction

Volatile organic compounds (VOCs) from industrial activities pose significant environmental risks due to their toxicity and their role in forming secondary pollutants, including secondary organic aerosols (SOAs), photochemical smog, and ozone [1,2,3]. Aromatic hydrocarbons, a major subset of VOCs, are key contributors to SOA and ozone production through photochemical processes [4,5]. Despite their pollutant status, these carbon and hydrogen-based compounds hold potential value within the frameworks of a circular economy and green chemistry [6,7,8]. Therefore, integrating the decontamination and utilization of aromatic hydrocarbon pollutants can yield substantial economic and environmental benefits [9,10].

In theory, the pyrolysis of hydrocarbons (C_x_H_y_) presents a promising approach to meet this objective, yielding solid carbon materials and hydrogen gas through the following reaction (1). This process is a non-oxidative thermochemical decomposition reaction. When used to treat aromatic hydrocarbons, it can prevent their breakdown into H_2_O and CO_2_, thus advancing carbon neutrality and reducing carbon dioxide emissions [11,12,13]. Indeed, due to methane’s high H:C ratio (4:1), plentiful supply, and cost-effectiveness in areas with natural gas resources, CH_4_ pyrolysis has been widely studied for CO_2_-free H_2_ generation and carbon material production, either independently or simultaneously [14,15,16,17,18,19]. Consequently, it has become one of the most potential-rich hydrocarbon pyrolysis processes. Diverse methods, including heterogeneous catalytic, plasma, and molten metal processes, have been explored [20,21,22]. However, heterogeneous catalytic CH_4_ pyrolysis is regarded as a crucial process deserving substantial effort, particularly in minimizing catalyst deactivation and producing carbon as nanotubes (CNTs) and nanofibers (CNFs) [23].

(1) $$C_{x}H_{y}\to xC_{\left(s\right)}+(y/2)H_{2,\left(g\right)}$$

To reduce the cost of the process, the generation of high-value CNTs and CNFs could be beneficial. These materials have wide-ranging applications as adsorbents, catalyst supports, conductive polymers, and more, thanks to their outstanding physical, chemical, electrical, mechanical, and optical characteristics [24,25,26,27]. Carbon nanotubes (CNTs) and carbon nanofibers (CNFs) were first synthesized using Ni/Al_2_O_3_ catalysts for the catalytic decomposition of hydrocarbons by Baker et al. [28]. Since then, numerous studies have focused on optimizing Ni/Al_2_O_3_ catalyst composition, preparation methods, and reaction conditions to control the growth of CNTs and CNFs [29]. Recently, Lee et al. demonstrated a novel fluidized-bed reactor system utilizing a Ni/Al_2_O_3_ catalyst for the continuous production of high-quality CNTs with controlled diameter and length, offering a promising route for scalable CNT synthesis [30]. Furthermore, these catalysts have been studied for their ability to decompose various hydrocarbons into CNTs, including acetylene, ethylene, propylene, benzene, toluene, and hexane [31,32]. In our study, the Ni/Al_2_O_3_ catalyst was employed due to its remarkable activity and stability, demonstrating exceptional catalytic performance throughout the process. Zhang et al. investigated the influence of silica doping on hydrogen production from the pyrolysis and reforming of waste tires using a Ni-loaded alumina catalyst, as well as its impact on carbon deposition on the catalyst [33]. Additionally, Ni/Al_2_O_3_ catalysts are extensively utilized in synthesizing carbon materials via chemical vapor deposition (CVD). Kim et al. investigated the Cu-doped Ni/Al_2_O_3_ catalyst for CVD synthesis of carbon nanofibers [34]. Previous research has primarily concentrated on cost-effective production strategies for CO_X_-free hydrogen or high-quality carbon materials utilizing catalytic pyrolysis. In our investigation, we applied the Ni/Al_2_O_3_ catalyst for treating hydrocarbon VOCs; these VOCs were effectively removed and converted into hydrogen and carbon through catalytic pyrolysis processes aimed at achieving pollutant removal alongside resource recovery from conversion products.

In this study, we undertook a comprehensive investigation into the efficient conversion of aromatic hydrocarbons. The C-H bond energy of aromatic hydrocarbons is relatively low (316 kJ/mol), suggesting that they can be effectively removed and converted into CO_x_-free hydrogen and solid carbon under catalytic conditions, in contrast to methane (435 kJ/mol) [35,36]. Consequently, we propose that by selecting an appropriate catalyst and optimizing reaction conditions, it is feasible to achieve efficient removal and conversion of aromatic hydrocarbons. To this end, we synthesized a Ni/Al_2_O_3_ catalyst and assessed its performance in the thermochemical decomposition of toluene (as a representative model for aromatic hydrocarbons). The results indicated that under optimal conditions, the Ni/Al_2_O_3_ catalyst exhibited high performance, removing 1328 mg/g of toluene from the gas stream, generating 915 mg/g of carbon material, and producing 1234 mL/g of CO_x_-free hydrogen. These findings not only demonstrate the high efficiency of the Ni/Al_2_O_3_ catalyst but also provide a significant scientific foundation for developing and implementing new technologies aimed at treating volatile aromatic hydrocarbons. The contribution of this study is to offer innovative ideas and methodologies for advancing technology related to the conversion of aromatic hydrocarbons.

## 2. Results and Discussion

### 2.1. Comprehensive Characterization and Preparation of Ni/Al_2_O_3_ Catalyst for Toluene Pyrolysis

**Figure 1:**
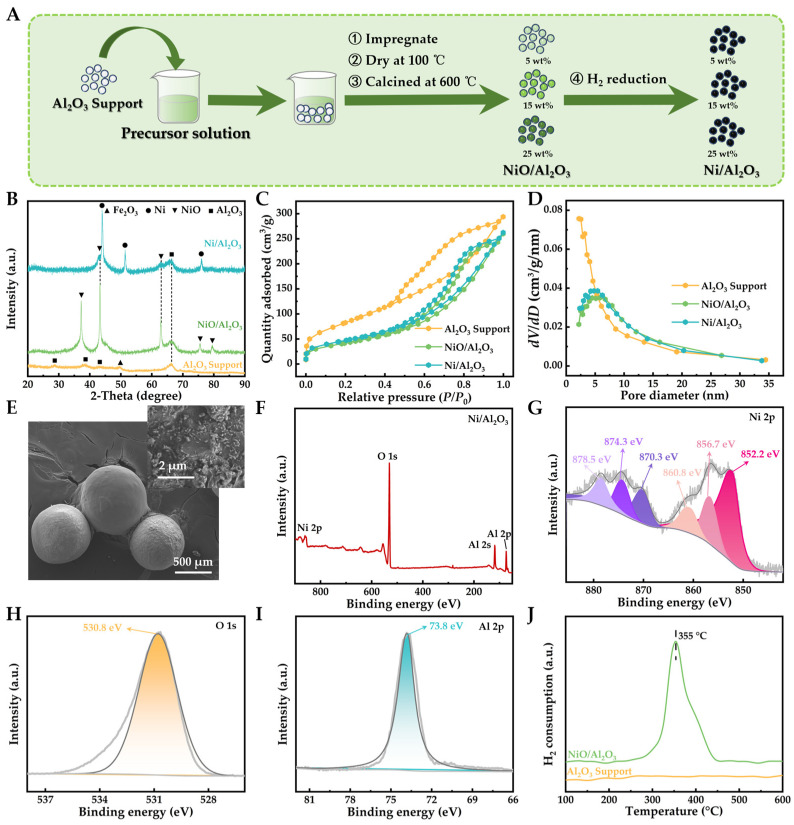
(**A**) Scheme of the experimental procedure of Ni/Al_2_O_3_. (**B**) XRD patterns, (**C**) adsorption–desorption isotherms of nitrogen, and (**D**) pore size distribution of Al_2_O_3_ support, NiO/Al_2_O_3_, and Ni/Al_2_O_3_. (**E**) SEM image of Ni/Al_2_O_3_. (**F**) XPS survey spectra, (**G**) Ni 2p, (**H**) O 1 s, and (**I**) Al 2p high-resolution XPS spectra of Ni/Al_2_O_3_. (**J**) H_2_-TPR profiles of Al_2_O_3_ support and NiO/Al_2_O_3_.

The preparation process of the Ni/Al_2_O_3_ catalyst is illustrated in Figure 1A, showcasing images of the catalyst, its NiO/Al_2_O_3_ precursor, and the initial raw materials (Al_2_O_3_ and Ni(NO_3_)_2_ solution). The catalyst maintains a spherical morphology and exhibits a gray–black appearance due to the Ni(II) loading and subsequent chemical transformation. Stoichiometric analysis indicated that the prepared catalyst had a Ni loading of 15% by weight. The Al_2_O_3_ was identified as low-crystallinity gamma-alumina (*γ*-Al_2_O_3_; PDF Number 48-0366) based on the presence of weak peaks at 2*θ* angles of 27.8°, 38.2°, 43.8°, and 67.1°, as well as significant background noise in its spectrum (Figure 1B). The emergence of a minor peak at 49.5° can be attributed to the existence of trace Fe_2_O_3_ impurities within the acquired Al_2_O_3_. The Fe_2_O_3_ content in the Al_2_O_3_ support (≤0.04%) is remarkably low in comparison to the NiO loading and has negligible impact on the reduction of the NiO phase. Furthermore, the NiO anchored on Al_2_O_3_ produces diffraction signals at 37.2°, 43.3°, 62.9°, 75.4°, and 79.4° (Figure 1B), corresponding to the (111), (200), (220), (311), and (222) planes of NiO (PDF No. 47-1049; Cubic, *Fm-3m* space group, a = 0.418 nm) [37]. The NiO in the NiO/Al_2_O_3_ precursor is then transformed into Ni (PDF No. 01-1258; Cubic, *Fm-3m* space group, a = 0.354 nm) through hydrogen reduction (Figure 1B), yielding the Ni/Al_2_O_3_ catalyst for toluene catalytic pyrolysis [38].

Figure 1C,D present N_2_ adsorption–desorption isotherms and pore diameter distributions of Al_2_O_3_, NiO/Al_2_O_3_, and Ni/Al_2_O_3_. All three Al_2_O_3_-based materials show a type IV isotherm with an H2 hysteresis loop, indicating a mixed micro–mesoporous structure with tubular shapes and granular interstices. Al_2_O_3_ exhibits a larger specific surface area and micropore volume compared to NiO/Al_2_O_3_ and Ni/Al_2_O_3_, as well as distinct pore size distribution and smaller pore sizes (Figure 1C,D and Table S1). Furthermore, low-magnification scanning electron microscopy (SEM) images reveal that the Ni/Al_2_O_3_ catalyst consists of spherical particles with well-defined shapes (Figure 1E). A high-magnification SEM image exhibits irregular fine particles attributed to the nickel loading on Al_2_O_3_ (Figure 1E). To investigate the dispersion of nickel particles, a transmission electron microscopy (TEM) analysis of the Ni/Al_2_O_3_ catalyst was conducted. Figure S1 illustrates that the black-contrasted nickel nanoparticles are thoroughly embedded within the porous Al_2_O_3_ support. These observations suggest that NiO and Ni are predominantly distributed within the micropores or narrow mesopores of Al_2_O_3_, forming nano/micro-particles and generating interstitial pores through aggregation.

The elemental composition and chemical state of the Ni/Al_2_O_3_ catalyst were investigated using X-ray photoelectron spectroscopy (XPS). The full survey spectrum confirmed the presence of Ni, Al, and O in the catalyst (Figure 1F). In Figure 1G, the two peaks at 852.2 eV and 874.3 eV are attributed to Ni 2p 3/2 and Ni 2p 1/2, respectively, indicating the presence of elemental nickel in Ni/Al_2_O_3_. The peaks observed at 856.7 eV and 874.3 eV in the Ni 2p spectrum can be ascribed to the spin-orbit characteristics of nickel (II) and reflect the Ni^2+^ oxidation state resulting from surface oxidation, suggesting that a significant portion of NiO was effectively reduced to metallic nickel during hydrogen treatment, which is consistent with the corresponding X-ray diffraction (XRD) results presented in Figure 1B. The peaks at 860.8 eV and 878.5 eV correspond to their respective satellite features [39]. Additionally, the O 1s XPS spectrum showed a main peak at 530.9 eV, which can be ascribed to the lattice oxygen in Al_2_O_3_ (Figure 1H). The Al 2p XPS spectrum displayed a single peak at 73.8 eV, corresponding to Al (III) in the Al_2_O_3_ support (Figure 1I). These XPS results confirm the formation of metallic Ni nanoparticles on the Al_2_O_3_ support, consistent with the XRD analysis.

The reducibility of the NiO/Al_2_O_3_ precursor was evaluated by H_2_ temperature-programmed reduction (H_2_-TPR, Figure 1J). The TPR profile exhibited a main reduction peak centered at around 355 °C, attributed to the reduction of NiO to metallic Ni. The relatively high reduction temperature suggests a strong interaction between NiO and the Al_2_O_3_ support [40]. No reduction peaks were observed for the Al_2_O_3_ support itself, confirming that the reduction peak in the NiO/Al_2_O_3_ sample is solely due to the reduction of NiO species. The H_2_-TPR result indicates that the NiO/Al_2_O_3_ precursor can be effectively reduced to form the Ni/Al_2_O_3_ catalyst under the employed hydrogen reduction conditions.

The characterization results confirm the successful preparation of the Ni/Al_2_O_3_ catalyst, featuring well-dispersed Ni nanoparticles on a mixed micro–mesoporous Al_2_O_3_ support. The strong Ni-Al_2_O_3_ interaction and effective NiO reduction likely enhance its catalytic performance in toluene pyrolysis for decontamination and resource recovery.

### 2.2. Effect of Temperature on Pyrolytic Conversion of Toluene

**Figure 2:**
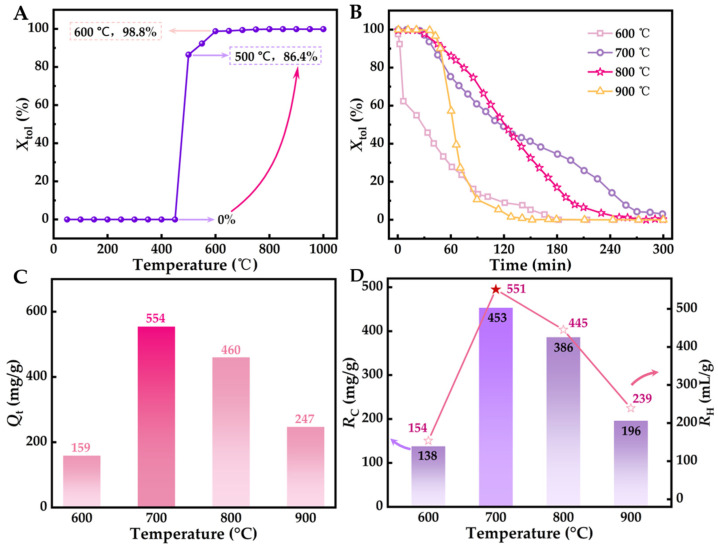
(**A**) Response curve of toluene’s temperature-dependent removal rate at 36.35 mg/L of *C*_in_, 0.205 L/min of *V*_g_, and 2.0 g of *m*_cat_. (**B**) Response curve of toluene’s time-dependent removal rate under different pyrolysis temperatures. (**C**) Total conversion capacity under different pyrolysis temperatures. (**D**) Carbon production and hydrogen production per unit mass of Ni/Al_2_O_3_ catalyst under different pyrolysis temperatures. Experimental conditions for B−D: *C*_in_ = 36.35 mg/L, *V*_g_ = 0.205 L/min, *m*_cat_ = 2.0 g.

Figure 2A demonstrates how the catalytic decomposition of toluene over a Ni/Al_2_O_3_ catalyst varies with temperature, under the specific conditions of *C*_in_ = 36.35 mg/L, *V*_g_ = 0.205 L/min, and *m*_cat_ = 2 g, showing a curve of the removal rate varying with temperature [41,42]. The observed curve, resembling an inverted “Z”, highlights the presence of an activation temperature range (450–600 °C) crucial for the Ni/Al_2_O_3_ catalyst’s activity in the toluene decomposition process. Below 450 °C, toluene does not decompose, while exceeding 600 °C results in near-complete decomposition (>98%). Within this activation range, the toluene conversion rate increases significantly with rising temperature. To further examine the influence of temperature on the Ni/Al_2_O_3_ catalyzed toluene conversion, reaction temperatures of 600 °C, 700 °C, 800 °C, and 900 °C were chosen for analysis under consistent conditions.

The curve showing the variation in toluene removal efficiency with temperature is displayed in Figure 2B, which aids in analyzing its pollution elimination behavior. The Ni/Al_2_O_3_ catalyst demonstrates high activity in toluene purification, as assessed by the *Q*_t_ values shown in Figure 2C. In one process, the activity of Ni/Al_2_O_3_ increases with rising temperature, indicated by the continuous increase in *Q*_t_ values. Conversely, another process reveals a decline in Ni/Al_2_O_3_ activity as the process progresses, closely related to temperature, with the slowest decrease observed at 700 °C. Therefore, the *Q*_t_ values enable quantitative analysis of the impact of temperature on the decline in Ni/Al_2_O_3_ activity, as illustrated in Figure 2D. The order of *Q*_t_ values is *Q*_t_ (700 °C) > *Q*_t_ (800 °C) > *Q*_t_ (900 °C) > *Q*_t_ (600 °C). The changes in Ni/Al_2_O_3_ activity during the catalytic pyrolysis of toluene may be linked to the deposition of solid carbon on its surface (detailed analysis below).

Figure 2D illustrates the relationship between the carbon production (*R*_C_) and hydrogen production (*R*_H_) per unit mass of the active Ni component in the Ni/Al_2_O_3_ catalyst during the pyrolysis of toluene at various temperatures. The results show that at 700 °C, *R*_C_ and *R*_H_ peak at 3021 mg/g and 3674 mL/g, respectively, aligning with the maximum adsorption capacity *Q*_t_ (3695 mg/g) observed at this temperature. This indicates that a mid–high temperature of around 700 °C is favorable for the efficient and selective catalytic decomposition of toluene into carbon and hydrogen by the Ni/Al_2_O_3_ catalyst. Therefore, subsequent experiments were conducted at 700 °C.

### 2.3. Effect of Process Conditions on Pyrolysis Conversion of Toluene

**Figure 3:**
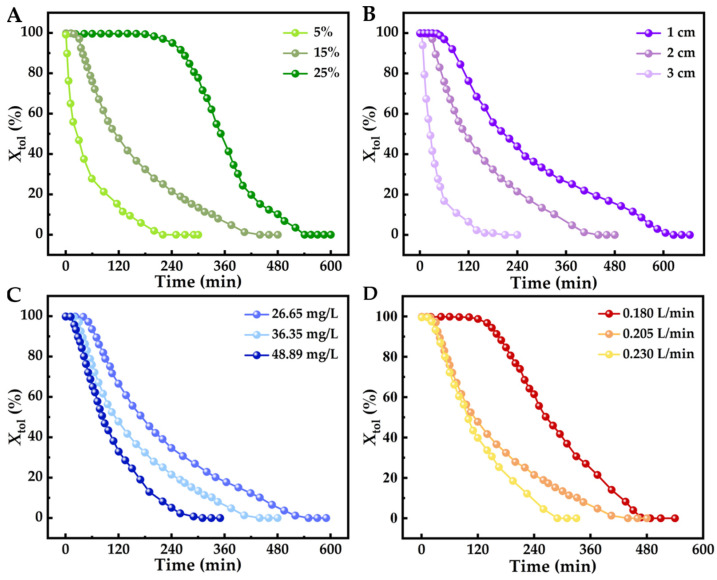
Response curve of toluene’s time-dependent removal rate at 800 °C under different (**A**) metal loading, (**B**) bed height, (**C**) inlet concentration, and (**D**) flow rate.

A reaction temperature of 700 °C was employed to study the impact of various operating parameters on toluene pyrolysis over a Ni/Al_2_O_3_ catalyst. These parameters included: active Ni loading, catalyst bed height, flow rate, and inlet toluene concentration. Figure 3A illustrates how increasing the Ni loading (5%, 15%, 25%) led to a corresponding improvement in toluene removal efficiency, under specific conditions of *C*_in_ = 36.35 mg/L, *V*_g_ = 0.205 L/min, and *m*_cat_ = 2 g. This trend of higher Ni content correlating with greater catalytic activity was further evidenced by the extended active durations observed (300 min, 480 min, 600 min). Furthermore, analysis of specific activity indicators (*Q*_t_, *R*_C_, *R*_H_) revealed a consistent positive correlation with increasing Ni loading, as detailed in Table 1.

The increase in Ni/Al_2_O_3_ catalyst bed height (1, 2, 3 cm) significantly enhanced toluene removal efficiency under *C*_in_ = 36.35 mg/L, *V*_g_ = 0.205 L/min, and *w*_Ni_ = 15% as shown in Figure 3B. This improvement is attributed to the prolonged contact time between toluene and the catalyst, resulting from the higher bed height, which fosters a more complete conversion. Additionally, a consistent positive correlation was observed between catalyst bed height and specific activity indicators (*Q*_t_, *R*_C_, *R*_H_), as presented in Table 1.

The impact of varying inlet toluene concentrations on removal efficiency is depicted in Figure 3C, where *V*_g_ = 0.205 L/min, *m*_cat_ = 2.0 g, and *w*_Ni_ = 15% were held constant. As the inlet concentration increased, a leftward shift in the decline of toluene removal efficiency was observed (Figure 3C). This suggests that while higher initial concentrations can be processed, the catalyst reaches saturation more rapidly. The observation that decontamination capacity per unit mass of catalyst (*Q*_t_) and resource recovery indicators (*R*_C_ and *R*_H_) decreased with higher inlet concentrations (Table 1) further supports this. These findings indicate that the Ni/Al_2_O_3_ catalyst demonstrates enhanced specific activity and prolonged stable operation under lower toluene concentration conditions.

Under controlled conditions (2 g catalyst, 36.35 mg/L inlet concentration, 15% *w*_Ni_), adjusting the flow rate affects both the frequency of toluene molecule exposure and contact time, evaluating Ni/Al_2_O_3_’s performance in toluene pyrolysis. Results show higher flow rates shift the toluene removal efficiency curve left and down (Figure 3D), decreasing *X*_tol_. However, increased flow can reduce residence time in the catalyst bed, potentially limiting toluene interaction with active sites. Metrics for specific activity (*Q*_t_, *R*_C_, *R*_H_) decrease with higher flow rates (Table 1), aligning with findings on varying inlet concentrations. This suggests contact time has a minor effect on toluene–Ni/Al_2_O_3_ interactions in the tested system. Optimizing these parameters is essential for improving toluene removal and maximizing the recovery of valuable byproducts like hydrogen and carbon nanostructures.

### 2.4. Re-Usability of a Ni/Al_2_O_3_ Catalyst

A straightforward physical method was employed to separate the deposited carbon from the Ni/Al_2_O_3_ catalyst, and the regenerated catalyst underwent a repeatable reuse test (Figure S2A). The findings indicate that as the cycle number increases, the removal rate curve of toluene for the Ni/Al_2_O_3_ catalyst exhibits a slight leftward shift (Figure S2A). Furthermore, after four consecutive uses, the *Q*_t_ value remains elevated (Figure S2B). These results demonstrate that simple physical separation can effectively regenerate carbon-coated Ni/Al_2_O_3_ catalysts while preserving a certain level of stability.

### 2.5. Structure of Deposited Carbon on a Ni/Al_2_O_3_ Catalyst

**Figure 4:**
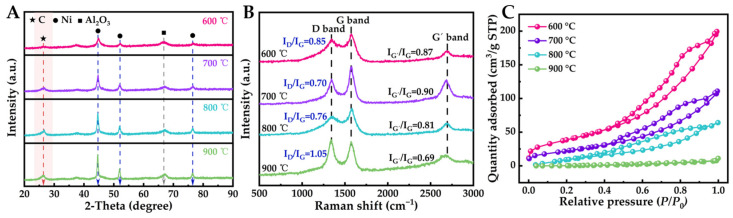
(**A**) XRD spectra (**B**) Raman spectra and (**C**) N_2_ adsorption–desorption curves of carbon materials under different temperatures.

In the study of toluene catalytic pyrolysis using a Ni/Al_2_O_3_ catalyst at temperatures of 600 °C, 700 °C, 800 °C, and 900 °C, the process predominantly generated solid carbon and hydrogen. XRD analysis of the carbon revealed a significant peak at a 2*θ* value of 26.5°, characteristic of graphitic carbon with hexagonal symmetry (Figure 4A, PDF No. 12-0212; *P63/mmc* space group, lattice parameters a = b = 0.246 nm and c = 0.674 nm). Additionally, the XRD spectra identified four minor peaks at 2*θ* values of 44.7°, 51.3°, and 76.6°, corresponding to Ni (PDF No. 01-1258; cubic, *Fm-3m* space group, a = 0.354 nm). A further peak at 67.0° was attributed to *γ*-Al_2_O_3_ impurities, likely introduced during the handling of the solid carbon samples.

The presence of a graphitic carbon structure in the deposited carbon indicates the formation of well-ordered, crystalline carbon nanostructures during the catalytic pyrolysis of toluene. The graphitic nature of the carbon suggests a high degree of sp^2^ hybridization and a layered structure, characteristic of CNFs or CNTs. The formation of these graphitic carbon nanostructures can be attributed to the catalytic effect of Ni nanoparticles, which serve as nucleation sites for carbon growth.

The mechanism of carbon nanostructure formation during the catalytic pyrolysis of toluene involves several steps. Firstly, toluene molecules adsorb onto the Ni nanoparticle surface and undergo catalytic decomposition, breaking down into smaller carbon-containing species such as methyl and phenyl radicals. These radicals then diffuse on the Ni nanoparticle surface and interact, leading to the nucleation and growth of graphitic carbon layers. As the process continues, the graphitic layers stack and extend, forming elongated nanofibers or nanotubes.

The weak signal peaks corresponding to Ni and *γ*-Al_2_O_3_ impurities in the XRD spectra of the deposited carbon are likely due to the physical separation process used to collect the carbon (Figure 4A). During this separation, small fragments of the Ni/Al_2_O_3_ catalyst may have been inadvertently included in the carbon sample, leading to the detection of these impurities.

The Raman spectra of carbon materials at varying temperatures further corroborate the findings from XRD and microscopic observations (Figure 4B). The presence of D, G, and G’ bands in the Raman spectrum is characteristic of graphitic carbon materials. The D band corresponds to a mode associated with defects or structural disorder, indicating the existence of such imperfections within the carbon material. The G band arises from the vibrations of sp^2^ carbon atoms in-plane and serves as an indicator of graphitic order. The G’ band represents the second harmonic of the D band and is indicative of sp^2^ hybridized carbon. The relative intensities of these bands yield insights into the purity and crystallinity of the carbon material [43].

In comparison to carbon materials synthesized at 600 °C, 800 °C, and 900 °C, those produced at 700 °C exhibit a lower ID/IG ratio alongside a higher IG/IG’ ratio, suggesting enhanced purity and crystallinity. A reduced ID/IG ratio signifies decreased ordering in graphene along with an increased proportion of sp^2^ carbon atoms, reflecting a heightened state of sp^2^ hybridization. Conversely, an elevated IG/IG’ ratio indicates greater degrees of graphitization and more orderly stacking among graphene layers. These results align with XRD analysis outcomes as well as subsequent SEM observations, confirming that the carbon material obtained at a moderate temperature of 700 °C possesses a more crystalline and ordered structure.

The nitrogen adsorption–desorption isotherms of the carbon materials display mesoporous characteristics, with type IV isotherms and H3 hysteresis loops (Figure 4C, Table S2). The presence of mesopores in the carbon materials can be attributed to the gaps between entangled nanofibers or voids generated by irregular stacking of graphene layers. As the pyrolysis temperature increases, the specific surface area and pore volume of the carbon materials decrease, which can be explained by the higher degree of graphitization and denser structure at higher temperatures.

**Figure 5:**
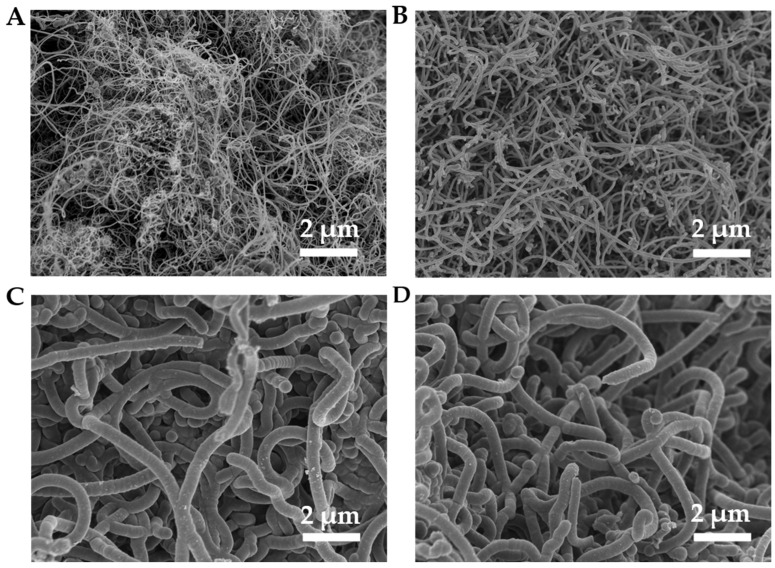
SEM results of carbon materials at (**A**) 600 °C, (**B**) 700 °C, (**C**) 800 °C, and (**D**) 900 °C.

The morphology of the products was observed using SEM. Interestingly, we found that reaction temperature significantly influences their morphology. Specifically, at 600 °C and 700 °C, the carbon materials formed by the catalytic pyrolysis of toluene exhibit diameters of a few nanometers, appearing as entangled and elongated filaments (Figure 5A,B). At 800 °C and 900 °C, the carbon materials have diameters of several hundred nanometers, presenting as bent, short fibrous shapes (Figure 5C,D). It is speculated that these changes in solid carbon morphology result from the combined effects of reaction temperature on the catalytic conversion rate of toluene to carbon, as well as the subsequent rates of carbon nucleation and growth. Compared to temperatures of 600 °C, 800 °C, and 900 °C, the morphology of the carbon products at 700 °C is more refined. Additionally, the intensity of the diffraction peak on the (002) plane in its XRD spectrum is enhanced, providing mutual corroboration for these observations.

The carbon materials prepared by the pyrolysis of toluene on a Ni/Al_2_O_3_ catalyst exhibit a mesoporous structure and high specific surface area, offering significant application potential. Mesoporous carbon materials with high surface areas are ideal for various applications such as adsorption, catalysis, energy storage, and electrochemical devices. The presence of mesopores facilitates the diffusion and transport of molecules, ions, or electrolytes, while the high surface area provides abundant active sites for adsorption or catalytic reactions. The graphitic nature of the carbon materials also enhances their electrical conductivity and thermal stability, making them suitable for supercapacitors, lithium-ion batteries, and fuel cells.

## 3. Materials and Methods

### 3.1. Regents and Material

Nickel nitrate hexahydrate (Ni(NO_3_)_2_·6H_2_O), the nickel precursor, was supplied by Tianjin Damao Chemical Reagent Co., Ltd. (Tianjin, China). Toluene (99.5%) was sourced from Tianjin Kemiou Reagent Co., Ltd. (Tianjin, China). These analytical-grade reagents were used without further purification. The Al_2_O_3_ support (0.5–1 mm diameter) was procured from Shandong Runze Environmental Protection Material Co., Ltd. (Shandong, China). High-purity nitrogen (99.9%) and hydrogen (99.9%) gases (obtained from Xisanjiao Practical Gas Co., Ltd., Shijiazhuang, Hebei, China) were used for the catalytic pyrolysis experiments and catalyst preparation.

### 3.2. Preparation of the NiO/Al_2_O_3_ Precursor

The NiO/Al_2_O_3_ precursor, used for the 15 wt% Ni/Al_2_O_3_ catalyst, was prepared via a wet impregnation method. Ni(NO_3_)_2_·6H_2_O served as the nickel source, and *γ*-Al_2_O_3_ was used as the catalyst support. In a typical procedure, 17.5 g of Ni(NO_3_)_2_·6H_2_O was dissolved in 20 mL of deionized water to form a clear green solution (5% wt%, 5.2 g Ni(NO_3_)_2_·6H_2_O; 15% wt%, 17.5 g Ni(NO_3_)_2_·6H_2_O; 25% wt%, 33.2 g Ni(NO_3_)_2_·6H_2_O). Then, 20 g of *γ*-Al_2_O_3_ was added to the solution, and the mixture was stirred continuously at 50 °C for 4 h to ensure uniform distribution of the Ni precursor on the support. After impregnation, the mixture was dried in an oven at 100 °C for 12 h to remove any residual moisture. The resulting powder was placed in a quartz boat and calcined in a tube furnace at 600 °C for 4 h with a heating rate of 10 °C/min in an air atmosphere. During calcination, the Ni(NO_3_)_2_·6H_2_O precursor decomposed and formed NiO nanoparticles, which were well-dispersed on the surface of the *γ*-Al_2_O_3_ support. After naturally cooling to room temperature, the calcined sample was collected and stored in an airtight container for further use as the NiO/Al_2_O_3_ precursor.

### 3.3. Catalytic Pyrolysis of Toluene in a Ni/Al_2_O_3_ Filled Bed

A 2 g sample of the NiO/Al_2_O_3_ precursor was centrally placed in a fixed-bed flow column reactor. This reactor, featuring a quartz tube with a 12 mm inner diameter and 460 mm height, was situated within a resistance furnace. Quartz cotton sealed the reactor ends before use. The NiO/Al_2_O_3_-filled reactor was heated from ambient temperature to 500 °C at 20 °C/min under a 50 mL/min flow of a 10% H_2_/N_2_ reducing gas mixture, and held at this temperature for 20 min. Subsequently, the gas was switched to pure H_2_ and maintained for an additional hour to fully reduce NiO to metallic nickel. The Ni/Al_2_O_3_ catalyst was then cooled to room temperature under hydrogen protection. The temperature of the Ni/Al_2_O_3_ catalyst bed was adjusted according to the required heating mode (elevating from room temperature to 1000 °C at 20 °C/min) or to specific target temperatures (600 °C, 700 °C, 800 °C, or 900 °C). Immediately afterward, a simulated toluene gas with the inlet concentration (26.65 mg/L, 36.35 mg/L, or 48.89 mg/L) and flow rate (0.180 L/min, 0.205 L/min, or 0.230 L/min) was introduced, generated by bubbling nitrogen through liquid toluene in a constant temperature bath. Real-time toluene concentration was measured using a gas chromatograph (GC9790; Zhejiang Fuli Analytical Instrument Co., Ltd. (Zhejiang, China)) equipped with a hydrogen flame ionization detector, while pyrolysis gaseous products were analyzed with a GS-9000 gas analyzer (Tengzhou Lunan Xinke Instrument Co., Ltd. (Shandong, China)).

In this study, the Ni/Al_2_O_3_ catalyst demonstrated beneficial effects in three key areas: eliminating VOC pollution, producing value-added solid carbon, and generating oxygen-free hydrogen. These effects were quantified by toluene conversion rate (*X*_tol_, %), total conversion capacity (*Q*_t_, mg/g), carbon production per unit mass of active Ni component in the catalyst (*P*_C_, mg/g), and hydrogen production (*P*_H_, mL/g). The definitions of these metrics are provided in Equations (2)–(5)

(2) $$X_{\mathrm{tol}}=\frac{C_{\mathrm{in}}-C_{\mathrm{out},t}}{C_{\mathrm{in}}}\times 100\%$$

(3) $$Q_{t}=\frac{V_{g}\cdot C_{\mathrm{in}}}{m_{\mathrm{cat}}}\int_{0}^{t}\left(1-\frac{C_{\mathrm{out},t}}{C_{\mathrm{in}}}\right)dt$$

(4) $$R_{C}=\frac{m_{C}}{m_{\mathrm{cat}}}$$

(5) $$R_{H}=\frac{V_{g}\cdot 1000\cdot Y_{H}\cdot t}{m_{\mathrm{cat}}}$$

where the variables *C*_in_ and *C*_out,t_ represent the toluene concentrations at the inlet and outlet (mg/L) at any reaction time *t* (min); *V*_g_ is the gas flow rate (L/min) under standard conditions (298 K, 101.3 kPa); and *m*_cat_ is the mass of the catalyst (g). The total conversion capacity “*Q*_t_” refers to the cumulative toluene converted per unit mass of catalyst over the entire pyrolysis process, up to complete catalyst deactivation. *Y*_H_ denotes the average hydrogen percentage in the outlet gas (%), measured at intervals using a 1 L gas bag; *m*_C_ is the total carbon mass produced during pyrolysis (mg).

### 3.4. Reuse Experiment of a Ni/Al_2_O_3_ Catalyst

In the toluene thermal decomposition experiment conducted at *C*_in_ = 36.35 mg/L, *V*_g_ = 0.205 L/min, *m*_cat_ = 2 g, and *T* = 700 °C, fresh Ni/Al_2_O_3_ particles became enveloped in loose carbon. This resulted in the formation of a binary mixture comprising carbon and Ni/Al_2_O_3_. Following the cooling of this binary mixture to room temperature and its transfer from the reactor to a ceramic evaporation dish, it was gently agitated with weighing paper before being sieved through a stainless-steel mesh with an aperture size of 0.3 mm (50 mesh) to effectively separate solid carbon from the Ni/Al_2_O_3_ catalyst. This procedure was repeated three times to ensure optimal separation efficiency. The separated Ni/Al_2_O_3_ catalyst was subsequently reintroduced into the reactor as per the methodology outlined in Section 2.3 for a second round of catalytic thermal decomposition using toluene. This operation was performed four times to assess the reusability of the Ni/Al_2_O_3_ catalyst.

### 3.5. Materials Characterization

A comprehensive characterization was performed on the fresh Ni/Al_2_O_3_ catalyst, the spent catalyst after toluene pyrolysis, and the deposited carbon materials to analyze their physicochemical properties, morphology, and structure. XRD was conducted using a Bruker D8 Advance diffractometer with Cu Kα radiation (λ = 1.5406 Å) to identify the crystalline phases. The XRD patterns were recorded within a 2*θ* range of 20–90° with a step size of 0.02° and a scanning speed of 2°/min. The Ni nanoparticle crystallite size was determined using the Scherrer equation, based on the broadening of the Ni (111) diffraction peak. The morphology and surface structure of the catalyst and carbon materials were examined via SEM with a Hitachi S-4800 microscope at an accelerating voltage of 5 kV. The samples were sputter-coated with a gold layer to enhance conductivity prior to SEM imaging. The microstructures and morphologies of the samples were examined utilizing TEM (Hitachi H-7650, Tokyo, Japan). Textural properties were assessed through nitrogen adsorption–desorption measurements at 77 K using a Kubo X1000 automatic pore analyzer. Samples were degassed at 120 °C for 2 h under vacuum to remove moisture and impurities before measurement. Specific surface area was calculated using the Brunauer–Emmett–Teller (BET) method, while pore size distribution was determined using the Barrett–Joyner–Halenda (BJH) method from the isotherm’s desorption branch. Raman spectroscopy, conducted with a Renishaw inVia Raman microscope at a laser excitation wavelength of 532 nm, was used to investigate the structural properties and graphitization of the deposited carbon. Spectra were collected in the 500–3000 cm^−1^ range with a resolution of 1 cm^−1^, and the I_D_/I_G_ ratio (intensity ratio of the D band to the G band) was calculated to assess the degree of graphitization. XPS analysis used a Thermo Fisher Scientific ESCALAB QXi spectrometer with a monochromatic Al Kα X-ray source, this was employed to determine the surface chemical composition and oxidation states of the catalyst and carbon materials. Survey spectra were recorded with a pass energy of 200 eV, and high-resolution spectra of specific elements (Ni 2p, Al 2p, O 1s, and C 1s) were acquired with a pass energy of 50 eV. The binding energies were calibrated using the C 1s peak at 284.8 eV and the Shirley background was used for all the XPS spectra. TPR analysis was performed to study the reducibility of the NiO/Al_2_O_3_ precursor and the NiO-Al_2_O_3_ interaction using a Tensor 2 instrument. A 40 mg sample was loaded into a quartz U-tube reactor and pretreated at 200 °C for 1 h under argon flow (30 mL/min) to eliminate moisture and impurities. After cooling to room temperature, the sample was heated from 100 °C to 600 °C at 10 °C/min under a 10% H_2_/Ar flow (50 mL/min), with hydrogen consumption monitored via a thermal conductivity detector (TCD). Thermogravimetric analysis (TGA) was used to determine the carbon content of the spent catalyst and deposited carbon materials in an air atmosphere, using a TA Instruments Q600 SDT analyzer. Samples (10–15 mg) were heated from room temperature to 900 °C at 10 °C/min under air flow (100 mL/min).

## 4. Conclusions

This study introduces a novel approach using a Ni/Al_2_O_3_ catalyst for the efficient removal and conversion of volatile aromatic hydrocarbons, particularly toluene, into valuable products. Characterization of the catalyst using advanced techniques such as scanning electron microscopy, X-ray diffraction, and N_2_ adsorption–desorption isotherms reveals a spherical structure, well-dispersed Ni nanoparticles, and strong Ni-Al_2_O_3_ interactions, resulting in high catalytic activity. The research demonstrates that the Ni/Al_2_O_3_ catalyst effectively decomposes toluene into hydrogen and solid carbon materials, predominantly in the form of carbon nanofibers, with CO_x_-free hydrogen production. A moderate temperature of 700 °C was found to be most favorable for the catalytic process. Under optimized conditions, the Ni/Al_2_O_3_ catalyst removed 1328 mg/g of toluene, generated 915 mg/g of carbon material, and produced 1234 mL/g of hydrogen. The synthesized carbon nanofibers, characterized by a mesoporous structure and high specific surface area, hold potential application value in adsorption, catalysis, and energy storage. This integrated approach provides a promising pathway for an environmentally friendly reduction in VOC pollution.

## Figures and Tables

**Table 1:** Parameter calculation results under different process conditions.

Reaction Condition		*R*_C_ (mg/g)	*R*_H_ (mL/g)	*Q*_t_ (mg/g)
Metal loading (%)	5	191	169	180
	15	453	551	554
	25	915	1234	1328
Bed height (cm)	1	280	254	247
	2	453	551	554
	3	510	723	716
Inlet Concentration (mg/L)	26.65	475	556	579
	36.35	453	551	554
	48.89	450	481	512
Flow Rate (L/min)	0.180	683	753	918
	0.205	453	551	554
	0.230	346	455	486

## Data Availability

Data were contained within the article. The data presented in this study are available.

## Supplementary Materials

The following supporting information can be downloaded at: https://www.mdpi.com/article/10.3390/molecules29204868/s1, Table S1: Specific surface area, pore volume, and average pore diameter of the catalyst; Table S2: Specific surface area, pore volume, and average pore diameter of carbon materials. Figure S1. TEM image of Ni/Al_2_O_3_ catalyst. Figure S2. (A) Response curve of toluene’s time-dependent removal rate and (B) total conversion capacity for 4 consecutive regenerations. Experimental conditions: *T* = 700 °C, *C*_in_ = 36.35 mg/L, *V*_g_ = 0.205 L/min, *m*_cat_ = 2.0 g.

## References

[1] He C., Cheng J., Zhang X., Douthwaite M., Pattisson S., Hao Z. (2019). Recent advances in the catalytic oxidation of volatile organic compounds: A review based on pollutant sorts and sources. Chem. Rev..

[2] Zhang H., Wang Z., Wei L., Liu Y., Dai H., Deng J. (2024). Recent progress on VOC pollution control via the catalytic method. Chin. J. Catal..

[3] Wu P., Jin X., Qiu Y., Ye D. (2021). Recent progress of thermocatalytic and photo/thermocatalytic oxidation for VOCs purification over manganese-based oxide catalysts. Environ. Sci. Technol..

[4] Liu B., Ji J., Zhang B., Huang W., Gan Y., Leung D.Y.C., Huang H. (2022). Catalytic ozonation of VOCs at low temperature: A comprehensive review. J. Hazard. Mater..

[5] Ma X., Zhao D., Qian J., Ma Z., Cui J. (2022). Utilization of hematite particles for economical removal of *o*-xylene in a high-temperature gas-solid reactor. Molecules.

[6] Shi Y., Li Z., Wang J., Zhou R. (2021). Synergistic effect of Pt/Ce and USY zeolite in Pt-based catalysts with high activity for VOCs degradation. Appl. Catal. B Environ..

[7] He F., Muliane U., Weon S., Choi W. (2020). Substrate-specific mineralization and deactivation behaviors of TiO_2_ as an air-cleaning photocatalyst. Appl. Catal. B Environ..

[8] Liu Y., Zhao Z., Wang Q., Wang T., Zhong L., Pan W.-P. (2024). Insights into the removal of volatile organic compounds by three-dimensional ordered microporous zeolite-templated carbon: A combined experimental study and density functional theory calculation. Chem. Eng. J..

[9] Ma M., Gao K., Ma Z., Ding J. (2021). Influence of preparation method on the adsorptive performance of silica sulfuric acid for the removal of gaseous o-xylene. Sep. Purif. Technol..

[10] Gao K., Ma M., Liu Y., Ma Z. (2021). A comparative study of the removal of o-xylene from gas streams using mesoporous silicas and their silica supported sulfuric acids. J. Hazard. Mater..

[11] Deng H., Li K., Li H., Li X., Zhang L., Cao W. (2011). Densification behavior and microstructure of carbon/carbon composites prepared by chemical vapor infiltration from xylene at temperatures between 900 and 1250 °C. Carbon.

[12] Guo Y., Wen M., Song S., Liu Q., Li G., An T. (2022). Enhanced catalytic elimination of typical VOCs over ZnCoOx catalyst derived from in situ pyrolysis of ZnCo bimetallic zeolitic imidazolate frameworks. Appl. Catal. B Environ..

[13] Xiang W., Zhang X., Chen K., Fang J., He F., Hu X., Tsang D.C.W., Ok Y.S., Gao B. (2020). Enhanced adsorption performance and governing mechanisms of ball-milled biochar for the removal of volatile organic compounds (VOCs). Chem. Eng. J..

[14] Sorcar S., Rosen B.A. (2023). Methane pyrolysis using a multiphase molten metal reactor. ACS Catal..

[15] Kang D., Palmer C., Mannini D., Rahimi N., Gordon M.J., Metiu H., McFarland E.W. (2020). Catalytic methane pyrolysis in molten alkali chloride salts containing iron. ACS Catal..

[16] Prabowo J., Lai L., Chivers B., Burke D., Dinh A.H., Ye L., Wang Y., Wang Y., Wei L., Chen Y. (2024). Solid carbon co-products from hydrogen production by methane pyrolysis: Current understandings and recent progress. Carbon.

[17] Patlolla S.R., Katsu K., Sharafian A., Wei K., Herrera O.E., Mérida W. (2023). A review of methane pyrolysis technologies for hydrogen production. Renew. Sustain. Energy Rev..

[18] Patlolla S.R., Sharafian A., Mérida W. (2024). Characterization of solid carbon from hydrocarbon pyrolysis in molten aluminum. Carbon.

[19] Ji Y., Palmer C., Foley E.E., Giovine R., Yoshida E., Sebti E., Patterson A.R., McFarland E., Clément R.J. (2023). Valorizing the carbon byproduct of methane pyrolysis in batteries. Carbon.

[20] Gadd G.E., Blackford M., Moricca S., Webb N., Evans P.J., Smith A.M., Jacobsen G., Leung S., Day A., Hua Q. (1997). The world’s smallest gas cylinders?. Science.

[21] Chen P., Wu X., Lin J., Tan K.L. (1999). High H_2_ uptake by alkalidoped carbon nanotubes under ambient pressure and moderate temperatures. Science.

[22] Rodriguez N.M., Kim M.-S., Baker R.T.K. (1994). Carbon nanofibers: A unique catalyst support medium. J. Phys. Chem..

[23] Kovalenko G.A., Rudina N.A., Chuenko T.V., Ermakov D.Y., Perminova L.V. (2009). Synthesis of catalytic filamentous carbon by the pyrolysis of alkanes on alumina–silica foam supporting nickel nanoparticles. Carbon.

[24] Zhang K., Huang Z., Yang M., Liu M., Zhou Y., Zhan J., Zhou Y. (2023). Recent progress in melt pyrolysis: Fabrication and applications of high-value carbon materials from abundant sources. SusMat.

[25] Zeng Y., Zhong J., Feng F., Ye D., Hu Y. (2024). Synergistic photothermal catalytic oxidation of methanol and toluene mixture over Co-MOFs-derived catalyst: Interfacial and promotion effects. Chem. Eng. J..

[26] Hoecker C., Smail F., Pick M., Boies A. (2017). The influence of carbon source and catalyst nanoparticles on CVD synthesis of CNT aerogel. Chem. Eng. J..

[27] Shandakov S.D., Kosobutsky A.V., Rybakov M.S., Sevostyanov O.G., Russakov D.M., Lomakin M.V., Vershinina A.I., Chirkova I.M. (2018). Effect of gaseous and condensate products of ethanol decomposition on aerosol CVD synthesis of single-walled carbon nanotubes. Carbon.

[28] Baker K., Harris P., Thomas R., Waite R. (1973). Formation of filamentous carbon from iron, cobalt and chromium catalyzed decomposition of acetylene. J. Catal..

[29] Serp P., Castillejos E. (2010). Catalysis in carbon nanotubes. ChemCatChem.

[30] Bae K., Kim D., Dung P.A., Lee D., Hwang B., Go K.S., Kim W., Lee J.K., Im J.S., Kang S.C. (2024). Simultaneous and continuous production of carbon nanotubes and hydrogen by catalytic CH_4_ decomposition in a pressurized fluidized-bed reactor. Ind. Eng. Chem. Res..

[31] Hernadi K., Fonseca A., Nagy J.B., Siska A., Kiricsi I. (2000). Production of nanotubes by the catalytic decomposition of different carbon-containing compounds. Appl. Catal. A Gen..

[32] Ermakova M.A., Ermakov D.Y., Kuvshinov G.G. (2000). Effective catalysts for direct cracking of methane to produce hydrogen and filamentous carbon. Appl. Catal. A.

[33] Zhang Y., Tao Y., Huang J., Williams P. (2017). Influence of silica–alumina support ratio on H_2_ production and catalyst carbon deposition from the Ni-catalytic pyrolysis/reforming of waste tyres. Waste Manag. Res..

[34] Kim S., Kim K., Ahn H., Cho K. (2008). Characterization of graphitic nanofibers synthesized by the CVD method using nickel–copper as a catalyst. J. Alloys Compd..

[35] Schwarz H. (2011). Chemistry with methane: Concepts rather than recipes. Angew. Chem. Int. Ed. Engl..

[36] Szwarc M. (1948). The C–H bond energy in toluene and xylenes. J. Chem. Phys..

[37] Lv Y., Li J., Feng S., Liu P., Hao F., Xiong W., Luo H.A. (2018). Multi-walled carbon nanotubes supported nickel nanoparticles doped with magnesia and copper for adiponitrile hydrogenation with high activity and chemoselectivity under mild conditions. Chem. Eng. J..

[38] Kang Y., Wang W., Pu Y., Li J., Chai D., Lei Z. (2017). An effective Pd-NiO_x_ -P composite catalyst for glycerol electrooxidation: Co-existed phosphorus and nickel oxide to enhance performance of Pd. Chem. Eng. J..

[39] Damyanovaa S., Shterevaa I., Pawelecb B., Mihaylovc L., Fierro J. (2020). Characterization of none and yttrium-modified Ni-based catalysts for dry reforming of methane. Appl. Catal. B.

[40] Sun M., Xia J., Wang H., Liu X., Xia Q., Wang Y. (2018). An efficient Ni_x_Zr_y_O catalyst for hydrogenation of bio-derived methyl levulinate to *γ*-valerolactone in water under low hydrogen pressure. Appl. Catal. B Environ..

[41] Wang Y., Ma X., Wang H., Zhao D., Liu Y., Ma Z. (2024). Enhancement of gaseous *o*-xylene elimination by chlorosulfonic acid-modified H-zeolite socony mobil-5. Molecules.

[42] Zhao D., Liu Y., Ma X., Qian J., Ma Z. (2022). Reactive adsorption performance and behavior of gaseous cumene on MCM-41 supported sulfuric acid. Molecules.

[43] Li Z., Deng L., Kinloch I., Young R. (2023). Raman spectroscopy of carbon materials and their composites: Graphene, nanotubes and fibres. Prog. Mater. Sci..

