# Supplementary material for: Efficient Toluene Decontamination and Resource Utilization through Ni/Al2O3 Catalytic Cracking
Source: Molecules. 2024 Oct 14;29(20):4868. doi: 10.3390/molecules29204868 (PMC11510176; doi:10.3390/molecules29204868)
Supplement: Supplementary file 1 [file molecules-29-04868-s001.zip › molecules-3187075-supplementary.pdf]

Supplementary Information

Efficient Toluene Decontamination and Resource Utilization through Ni/Al_2O_3 Catalytic Cracking

Yifei Niu [1], Xiaolong Ma [2,*], Guangyi Lu [1], Dandan Zhao [1], and Zichuan Ma [1,*]

1 Hebei Key Laboratory of Inorganic Nano-Materials, College of Chemistry and Material Sciences, Hebei Normal University, Shijiazhuang 050024, China; niuyf@stu.hebtu.edu.cn (Y.N.); luguangyi1838@163.com (G.L.); zhaodd@stu.hebtu.edu.cn (D.Z.)

2 School of Environmental Science and Engineering, Hebei University of Science and Technology, Shijiazhuang 050018, China

* Correspondence: maxiaolong2410@hebust.edu.cn (X.M.); mazc@hebtu.edu.cn (Z.M.)

Table S1. Specific surface area, pore volume and average pore diameter of catalyst.

Samples	S_{BET} (m^2/g)	V_{micro} (cm^3/g)	V_{meso} (cm^3/g)	V_{tot} (cm^3/g)	D_{aver} (nm)
Al_2O_3 Support	308.0	0.13	0.32	0.45	3.0
NiO/Al_2O_3	156.5	0.06	0.34	0.40	5.1
Ni/Al_2O_3	173.8	0.07	0.34	0.41	4.8

Table S2. Specific surface area, pore volume and average pore diameter of carbon materials.

Samples	S_{BET} (m^2/g)	V_{micro} (cm^3/g)	V_{meso} (cm^3/g)	V_{tot} (cm^3/g)	D_{aver} (nm)
600 °C	143.7	0.06	0.25	0.31	4.0
700 °C	83.2	0.03	0.14	0.17	3.0
800 °C	65.6	0.01	0.09	0.10	2.5
900 °C	0.63	0.00	0.02	0.02	2.3

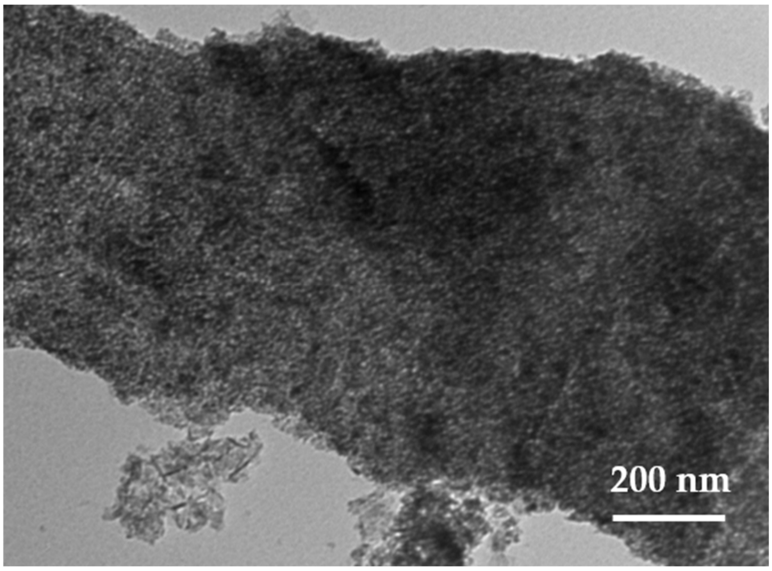

Figure S1. TEM image of Ni/Al_2O_3 catalyst.

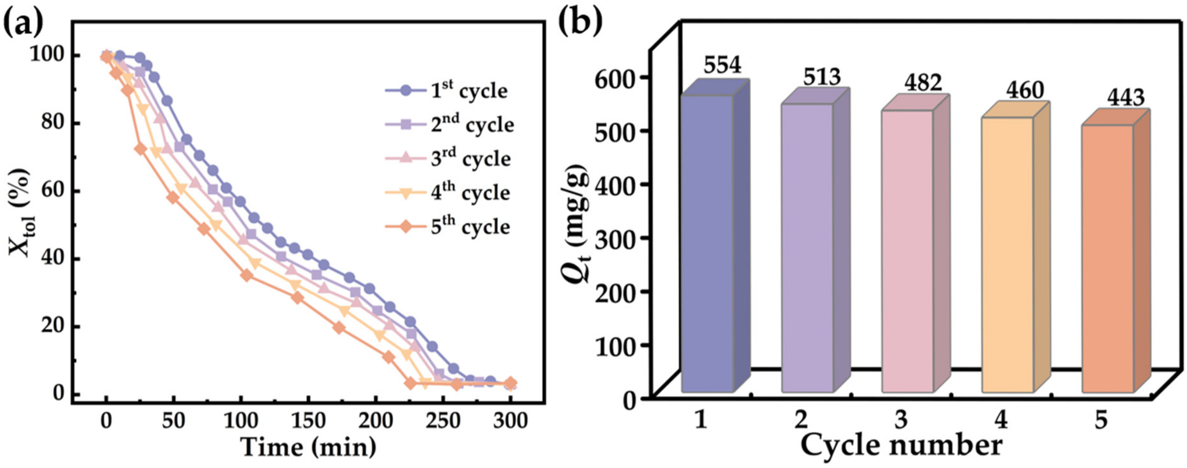

Figure S2. (A) Response curve of toluene's time-dependent removal rate and (B) total conversion capacity for 4 consecutive regenerations. Experimental conditions: T = 700 °C, C_{in} = 36.35 mg/L, V_g = 0.205 L/min, m_{cat} = 2.0 g.
